# Pentosan polysulfate inhibits *IL-1β*-induced *iNOS*, *c-Jun* and *HIF-1α* upregulation in canine articular chondrocytes

**DOI:** 10.1371/journal.pone.0177144

**Published:** 2017-05-04

**Authors:** Eugene C. Bwalya, Sangho Kim, Jing Fang, H. M. Suranji Wijekoon, Kenji Hosoya, Masahiro Okumura

**Affiliations:** Laboratory of Veterinary Surgery, Department of Veterinary Clinical Sciences, Graduate School of Veterinary Medicine, Hokkaido University, Sapporo, Hokkaido, Japan; SERGAS and IDIS, SPAIN

## Abstract

Osteoarthritic (OA) chondrocytes are shown to express inducible nitric oxide synthase (iNOS) which produces high concentrations of nitric oxide (NO), particularly when stimulated with proinflammatory cytokines. NO is involved in OA cartilage degradation. On the other hand, c-Jun N-terminal Kinase (JNK) pathway mediates the activation and transcription of c-Jun, which is required for interleukin-1 (IL-1)-induction of matrix metalloproteinases-13 (MMP-13) in OA pathogenesis. Therefore, the selective inhibition of iNOS and c-Jun is a promising target for treatment and prevention of OA. The purpose of the study was to investigate the inhibitory effects of pentosan polysulfate (PPS) on IL-1β-induced iNOS, c-Jun and HIF-α isoforms upregulation in canine articular chondrocytes (CACs). Primary (P0) chondrocytes were isolated and cultured from femoral head cartilages of three (3) dogs. First passage (P1) chondrocytes were preincubated with 0, 1, 5, 15 and 40 μg/mL of PPS for 4 hr before treatment with 10 ng/mL rhIL-1β for a further 8 hr. In addition, we evaluated the effects of single and multiple cytokine with or without LPS on iNOS protein induction. PPS significantly inhibited (*P* < 0.05) IL-1β-induced iNOS, c-Jun and HIF-1α mRNA upregulation in a dose-dependent pattern. iNOS mRNA was significantly inhibited at 15 and 40 μg/mL whereas c-Jun and HIF-1α were significantly downregulated at 5, 15 and 40 μg/mL of PPS compared to chondrocytes treated with only rhIL-1β. Intriguingly, CACs were recalcitrant to single IL-1β, TNF-α or LPS-induction of iNOS protein including to a combination of IL-1β+TNF-α, IL-1β+LPS except to TNF-α+LPS and IL-1β+TNF-α+LPS suggestive of a protective mechanism from iNOS detrimental effects on perpetuating OA. IL-1β+TNF-α+LPS-induced iNOS protein expression was significantly abrogated by PPS. We demonstrate for the first time that PPS is a novel inhibitor of IL-1β-induced iNOS, c-Jun, and HIF-1α mRNA upregulation and iNOS protein induction which may be beneficial for prevention and treatment OA.

## Introduction

Osteoarthritis (OA) is a degenerative joint disease that progressively causes loss of joint function [1] affecting not only articular cartilage but also involves the entire joint including the subchondral bone, ligaments, capsule, synovial membrane and menisci [2,3]. Osteoarthritic chondrocytes in affected joints have been shown to produce increased levels of inflammatory cytokines. Particularly, OA chondrocytes express inducible nitric oxide synthase (iNOS) and produce high concentrations of NO, especially upon stimulation by proinflammatory cytokines [4–7]. This pathologically increased NO production plays an important catabolic role in OA cartilage degradation. NO is partly responsible for the up-regulation of interleukin 1-beta-converting enzyme (ICE) and IL-18 synthesis while decreasing the level of the ICE inhibitor PI-9 [8]. There is also evidence indicating that NO plays a regulatory role in the activation of metalloproteinases in articular chondrocytes [4,9,10]. Furthermore, a relative deficit in the production of natural antagonists of the IL-1 receptor (IL-1Ra) has been reported in OA synovium and this has been associated to an excess production of NO. The excess production of NO combined with an upregulated IL-1 receptor level has been shown to be an additional enhancer of the catabolic effects of IL-1β in OA [8,11]. Therefore, the selective inhibition of pathologically enhanced NO synthesis has been identified as a promising novel therapeutic target for the prevention and treatment of inflammatory joint diseases [6,12–15]. The inhibition of iNOS by its natural inhibitors and selective agents has been shown to modulate the disease by reducing synovial inflammation and tissue damage [12,16–18]. As part of the signaling pathway, hypoxia inducible factor-2 alpha (HIF-2α) has been proposed as a catabolic factor that directly targets MMP-13 and iNOS through specific binding to the respective hypoxia-responsive elements [19–21]. However, the role of HIF-α isoforms (HIF-1α and HIF-2α) in OA pathogenesis is currently controversial and has led to species-dependent roles being proposed especially between murine and large mammals [22]. For example, HIF-2α has been shown by others to be responsible for hypoxic induction of cartilage matrix genes [22–25] and to be a potent regulator of autophagy in maturing mouse and human articular chondrocytes by acting as a brake to the autophagy accelerator function of HIF-1α [23].

Pentosan polysulfate (PPS), a semi-synthetic sulfated polysaccharide derived from wood of beech plant, *Fagus sylvatica* has been shown to improve synovial and subchondral blood flow, to limit cartilage matrix degeneration, and to stimulate hyaluronic acid and proteoglycan (PGs) synthesis [26–29]. Our laboratory previously showed its involvement in the prevention of inflammatory intracellular responses induced by IL-1β via inhibiting NFkB pathway and the phosphorylation of p38 and ERK but not JNK [30]. However, the JNK pathway has been shown to mediate the activation and transcription of c-Jun, which is required for IL-1-induction of MMP-13 [31]. Therefore, the inhibition of c-Jun is also a potential therapeutic target for the prevention and treatment of OA joints. Currently, the effect of PPS on iNOS, c-Jun and HIF-α isoforms in IL-1-stimulated articular chondrocytes remains unknown. Therefore, the objective of the present study was to investigate the effects of pentosan polysulfate (PPS) on IL-1-induced iNOS, c-Jun and HIF-α isoforms upregulation in canine articular chondrocytes (CACs). We hypothesized that PPS is a novel inhibitor of IL-1-induced iNOS and c-Jun upregulation in CACs.

## Materials and methods

### Chondrocytes culture

Canine articular cartilage samples were obtained with owners’ formal consent from femoral head cartilages of three dogs; a 1-year-old and 10-months-old that underwent femoral head and neck ostectomy due to Legg-Calvѐ-Perthes Disease (LCDP) and a 9-year-old that underwent hind limb amputation due to osteosarcoma affecting the distal femoral bone. The use of clinical samples and all samples from experimental dogs was in accordance with Hokkaido University Institutional Animal Care and Use Committee guidelines (approval #: 12–0059). Chondrocytes were released from cartilage by dissection and digestion for 24 hr in 0.3% Collagenase Type I (Wako Pure Chemicals Industries Ltd, Osaka, Japan) based on a protocol previously described elsewhere [32] with minor modifications. Primary (P0) chondrocytes were culture expanded to 85% confluence in 100-mm polystyrene culture plates (Corning, Lowell, MA, U.S.A.) containing 10% Fetal bovine serum (FBS; Nichirei Biosciences Inc, Tokyo, Japan) supplemented Dulbecco’s Modified Eagle’s Medium (DMEM; GIBCO BRL, Grand Island, NY, U.S.A.) containing, 10mM HEPES, 18mM NAHCO_3_, 100 U/mL Penicillin G potassium, and 73 U/mL Streptomycin sulphate (Wako Pure Chemical Industries). At confluence, P0 cells were detached using TryPle^TM^ Select Enzyme (1X) (GIBCO BRL). Cell count and viability was assessed by trypan blue exclusion test. First passage (P1) chondrocytes were used in this study.

### Chondrocytes treatment, preparation of cell extracts and analysis

Chondrocytes (P1) (7.0 x 10^5^ cells/well) were seeded in 12-well polystyrene culture plates (Corning) containing serum free (SF) DMEM and incubated at 37°C in 5% CO_2_ for 24 hr. Medium was removed and cells were treated either with 0 (control, CTL), 1, 5, 10 and 20 ng/mL recombinant human interleukin-1 beta (rhIL-1β) (Thermo Scientific, Life Technologies, Rockford, Illinois, USA) in SF DMEM for 8 hr or preincubated first with 1, 5, 15 and 40 μg/mL of PPS (Cartrophen Vet^®^ injection, NaPPS—100 mg/mL; Biopharm Australia, Bondi Junction, NSW, Australia) for 4 hr then treated with 10 ng/mL of rhIL-1β for a further 8 hr (Table 1).

**Table 1:** Treatment of first passage (P1) canine articular chondrocytes.

Treatment number	Treatment group	PPS (μg/mL)	rhIL-1β (ng/mL)
1	CTL	0	0
2	-	0	1
3	-	0	5
4	PC	0	10
5	-	0	20
6	PPS+rhIL-1β	1	10
7	PPS+rhIL-1β	5	10
8	PPS+rhIL-1β	15	10
9	PPS+rhIL-1β	40	10

PPS; pentosan polysulfate, rhIL-1β: recombinant human interleukin-1β, CTL; control without treatment, PC; positive control

In addition, the temporal effect of rhIL-1β on iNOS protein induction was evaluated by treating chondrocytes with 20 ng/mL of rhIL-1β for 0, 3, 6, 9 and 24 hr. Furthermore, to investigate the effects of multiple cytokines with or without lipopolysaccharide (LPS) (Wako Pure Chemicals Industries Ltd) on iNOS protein induction, chondrocytes were treated for 8 hr with single TNF-α (10 and 20 ng/mL) (Kingfisher Biotech, St Paul, Minnesota, USA) or LPS (50 and 75 μg/mL) (Wako Pure Chemicals Industries Ltd), and a combination of TNF-α (20 ng/mL) + LPS (50 μg/mL), LPS (25 μg/mL) + canine recombinant interleukin-1beta (20 ng/mL) (rcIL-1β, Kingfisher Biotech), TNF-α (20 ng/mL) + rcIL-1β (20 ng/mL), and rcIL-1β (20 ng/mL) + TNF-α (20 ng/mL) + LPS (50 μg/mL).

Total RNA and protein was extracted using TRIZol® reagent (Invitrogen, Life Technologies, Carlsbad, CA, USA) according to the manufacture’s protocol. Total RNA was quantified by spectrophotometry at 260 nm using Biowave DNA—WPA, 7123 V1.8.0 (Biochrom, Cambridge, UK) while protein was quantified by Bradford protein assay using the Thermo Scientific NanoDrop 2000c UV-Vis spectrophotometer (Thermo Scientific, NanoDrop products, Wilmington, Delaware, USA) and stored at -20°C until use.

Total of 500 ng RNA was reverse transcribed (RT) into cDNA using ReverTra Ace® qPCR RT Master Mix (Toyobo Co, Osaka, Japan) and amplified by PCR using TaKaRa Ex taq (TaKaRa Bio, Tokyo, Japan) according to manufacturer’s recommended protocol. This technique was employed to amplify mRNAs specific for iNOS, c-Jun, HIF-1α and HIF-2α. The PCR conditions were an initial denaturation of 94°C for 1 min followed by 35 cycles of 94°C for 30 s, 58°C for 30 s and 72°C for 30 s and then a finishing step of 72°C for 1 min. Gel electrophoresis was performed to detect mRNA bands. Quantitative real-time PCR (qPCR) was performed with KAPA SYBR® FAST qPCR kit (KAPA biosystems, Woburn, MA, U.S.A.) to determine the relative mRNA expression by the two step method. The qPCR conditions were an initial denaturation of 95°C for 20 s followed by 40 cycles of 95°C for 3 s and 60°C for 20 s then a pre-melt condition of 60°C for 90 s followed by a final melt step. The standard curve method was used to determine the relative mRNA quantification. All PCR reactions were validated by the presence of a single peak in the melt curve analysis and single band on gel electrophoresis. All mRNA expressions were normalised against the reference gene, glyceraldehyde-3-phosphate dehydrogenase (GAPDH) and the control (CTL) group was used as the calibrator to determine the mRNA-fold changes. The sequences, product size and accession codes for each of primers used in the experiments are indicated in Table 2. Primer sequences for all genes were designed using data published on the National Center for Biotechnology Information (NCBI) website using NCBI's standard and pairwise BLAST programs. For Western blot analyses, the identity of the proteins was verified by the expected protein band size relative to the protein marker or standard purified protein loaded as control. Because the antibodies used in our experiments were nonspecific to canine, to confirm immunogenic identity, homologous sequence comparison was performed using homology BLAST on NCBI website.

**Table 2:** Sequence of primers used in the experiments.

**Name of gene**	**Domain**	^a^**Primer**	^b^**Fragment**	**Accession**
**GAPDH**	664–683	5ˈ -CTGAACGGGAAGCTCACTGG-3ˈ	129 bp	NM_001003142.1
	773–792	5ˈ-CGATGCCTGCTTCACTACCT-3ˈ		
**iNOS**	13–32	5ˈ-TGGCAGTTTCTGTTCAAGGC-3ˈ	139 bp	XM_005624846.1
	132–151	5ˈ-TGCTGAGGCTGTGACACTTG-3ˈ		
**iNOS**	3681–3700	5ˈ- AATGGAGAGTTGGGCCTTCC-3ˈ	227 bp	NM_001313848.1
	3887–3907	5ˈ-TGGCCCTTAAGAGAAGACTGG-3ˈ		
**HIF-1α**	861–880	5ˈ-GTACTTCACTGCACAGGCCA-3ˈ	102 bp	NM_001287163.1
	943–962	5ˈ-ACAAATCAGCACCAAGCACG-3ˈ		
**HIF-2α**	1248–1267	5ˈ-TGCAAAGCACGGGGGCTACG-3ˈ	72 bp	XM_531807.3
	1300–1319	5ˈ-GGCTGCAGGTTGCGAGGGTT-3ˈ		
**c-Jun**	1144–1163	5ˈ-TCTACGACGATGCCCTCAAC-3ˈ	159 bp	XM_005620245.1
	1283–1302	5ˈ-TGAGCAGGTCCGAGTTCTTG-3ˈ		

^a^Primers for forward & reverse sense are presented in a 5ˈ to 3ˈ orientation

^b^The expected fragment size, GAPDH; glyceraldehyde-3-phosphate dehydrogenase, iNOS; inducible nitric oxide synthase, HIF; Hypoxia inducible factor

### Western blot: iNOS, HIF-1α and HIF-2α protein detection

The following primary antibodies were used; Rabbit polyclonal iNOS antibody (Thermo Fisher, Rockford, USA; Cat. #: PA1-036) (1: 100 dilution), mouse monoclonal Anti-HIF-1α Clone H1α67 (Sigma Aldrich, St. Louis, Missouri, USA; H6536) (1.0 μg/mL or 1: 1000 dilution) and EPAS-1 (HIF-2α) (C-16) goat polyclonal antibody (Santa Cruz Biotechnology, Dallas, USA; Cat. #: SC-8712) (1: 200 dilution). Normally, HIF-α isoforms protein are not readily detectable in human articular chondrocytes cultured under normoxic conditions as they are rapidly degraded in the presence of sufficient oxygen [33–37]. To verify that this is consistent with monolayer CACs cultured under normoxic conditions, the expression of HIF-1α and HIF-2α proteins was evaluated. F-Actin rabbit polyclonal antibody (Bioss Antibodies, Massachusetts, USA; Cat. #: 1571R) (1: 1000 dilution) was used as internal control. Secondary antibodies were; Pierce^®^ Goat anti-Rabbit Poly-HRP (Pierce Biotechnology, Rockford, Illinois, USA; Cat. #: 32260, Lot number–QG217308), Zymed^®^ Rabbit anti-mouse IgG-HRP conjugate (Invitrogen Corporation, Zymed Laboratories, Inc California, USA, Cat. #: 81–6720) and Anti-IgG goat Rabbit-Poly-HRP (R & D Systems, Minneapolis, Minnesota, USA, Cat. #: HAF0017). Western Blot Ultra-Sensitive HRP substrate (Takara Bio Inc., Otsu, Japan; Cat. #: T7104A, Lot #: AF3P025) was used for the signal generation. iNOS electrophoresis standard 130 kDa (Cayman Chemical, Michigan, USA, Cat. #: 360862) was used as standard iNOS control. Briefly, 20 μg of protein from each treatment was denatured and separated on 12% (w/v) sodium dodecyl sulfate-polyacrylamide gel (SDS-PAGE) for 55 min at 180 V in Tris/Glycine/SDS buffer (25 mM Tris, 190 mM Glycine, 0.1% SDS, pH 8.3). Two SDS PAGEs were run simultaneously, one was silver stained using AE-1360 EzStain Silver (Atto Corporation, Tokyo, Japan) according to manufacturer’s procedure to evaluate the expression profile of separated proteins while the proteins on other gel were electroblotted onto nitrocellulose membranes (Whatman, Dassel, Germany) at 60 V for 2 hr 30 minutes in transfer buffer containing 25 mM Tris, 190 mM Glycine, 20% methanol and 0.1% SDS (pH 8.3). Nonspecific antibody binding was blocked with 5% (w/v) skim milk prepared in Tris-buffered saline-Tween buffer (TBS-T, 20 mM Tris-HCl, 150 mM NaCl, 0.1% (w/v) Tween-20) for 1 hr at room temperature (RT) on a shaker. Membranes were then incubated in primary antibody at RT on a shaker for 1 hr then incubated overnight at 4°C in a refrigerator. Membranes were washed three (3) times with adequate TBST buffer at 5 min interval then incubated in HRP-conjugated secondary antibody at a dilution of 1:5,000 for 1 hr on a shaker at RT. Membranes were washed as before then incubated in HRP substrate for 5 min at RT. The protein-antibody reaction were visualized for chemiluminescent signal using FUJIFILM Luminescent Image Analyzer LAS-3000 (Fujifilm Life Science, LTD, Tokyo, Japan) according to the instrument manual for the imaging system. Protein bands were analysed using Multi-Gauge V 3.0 software (Fujifilm Life Science).

### Immunocytochemistry: Colocalization of PPS with c-Jun and NFkB p65

Immunocytochemistry (ICC) was performed to investigate colocalization of PPS with NFkB p65 and c-Jun, and to clarify whether PPS could inhibit NFkB p65 and c-Jun nuclear translocation. Chondrocytes (1 × 10^4^ cells) were plated in 8-well Permanox® slides (Thermo sciencetific nunc, New York, U.S.A.) in 500 μl of 10% FBS supplemented DMEM and incubated at 37°C in 5% CO_2_ for 24 hr. Cells were gently washed with 1 x PBS then preincubated in SF DMEM with or without 40 μg/mL of TRITC-PPS for 4 hr. Thereafter, 10 ng/mL of rcIL-1β was added to the medium with TRITC-PPS and cells incubated overnight at 37°C in 5% CO_2_. Cells were gently washed three times with cold 1 x PBS (2–8°C), fixed in cold methanol (-20°C) for 5 min at RT then washed on a shaker three times at 5min interval with cold 1 x PBS containing 0.1% tween 20 (PBST). Non-specific antibody binding was blocked by incubation for 1 hr in 5% bovine serum albumin (BSA) (Sigma-Aldrich, Lot # 019K1144) prepared in PBST. Primary antibodies used were anti-human c-Jun (H-79) Rabbit polyclonal antibody (Santa Cruz Biotechnology, SC-1694) (1:100 dilution) and anti-human NFkB p65 (C-20) Rabbit polyclonal antibody (Santa Cruz Biotechnology, SC-372) (1:100 dilution) by incubation at 4°C overnight. Cells were washed as before then incubated for 1 hr with goat anti-rabbit IgG-fluorescein isothiocyanate (FITC)-conjugated (Santa Cruz Biotechnology, SC-2012) (1:1000 dilution). Cells were washed as before and excess wash buffer removed. Prolong® Diamond Antifade Mountant with DAPI (Thermo Fisher Scientific, Eugene, OR, U.S.A) was used to stain the nucleus and mount the slides according to manufacturer’s instructions. Cells stained with only the secondary antibody were used as negative controls. Cells were viewed using Zeiss LSM 700 confocal laser microscope (Zeiss, Urbana, U.S.A).

### Data analysis

Quantitative qPCR data was analysed using SPSS version 16.0. Analysis of variance (ANOVA) was used to compare the mean relative gene expression between the treatments. Where significant difference was observed, multiple comparisons of group means was performed using *Post Hoc* Bonferroni. Linear regression analysis was performed to model the relationship between iNOS and HIF-α isoforms mRNA expression in response to IL-1 and PPS. Significance level was defined as *P* < 0.05. All quantitative results unless specified are presented as mean ± SD.

## Results

### Pentosan polysulfate (PPS) sodium inhibits IL-1β-induced iNOS, c-Jun and HIF-1α mRNA upregulation

**Fig 1:**
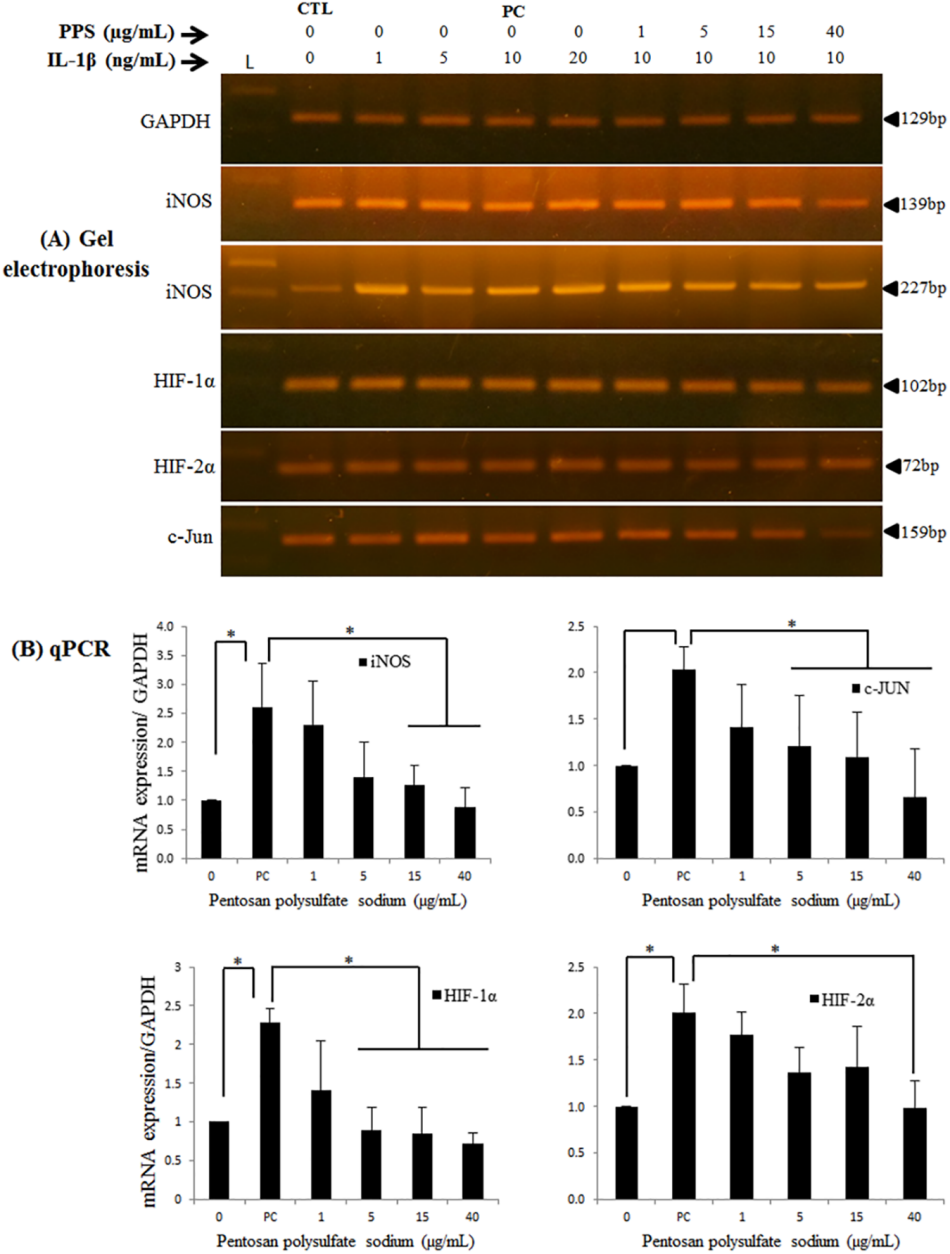
Pentosan polysulfate (PPS) inhibits rhIL-1β-induced iNOS, c-Jun and HIF-1α mRNA upregulation in canine articular chondrocytes. (A) As demonstrated by RT-PCR gel electrophoresis and (B) qPCR results, preincubation of chondrocytes with PPS inhibits rhIL-1β-induced iNOS, c-Jun and HIF-1α mRNA upregulation compared to the positive control (PC– 10 ng/mL rhIL-1β without PPS). L; 100bp DNA ladder, CTL; Control (no treatment), gel electrophoresis results shown are from one representative experiment of three (3) independent experiments, error bars represent the mean ± SD mRNA. Significant difference was defined as *P < 0.05.

**Fig 2:**
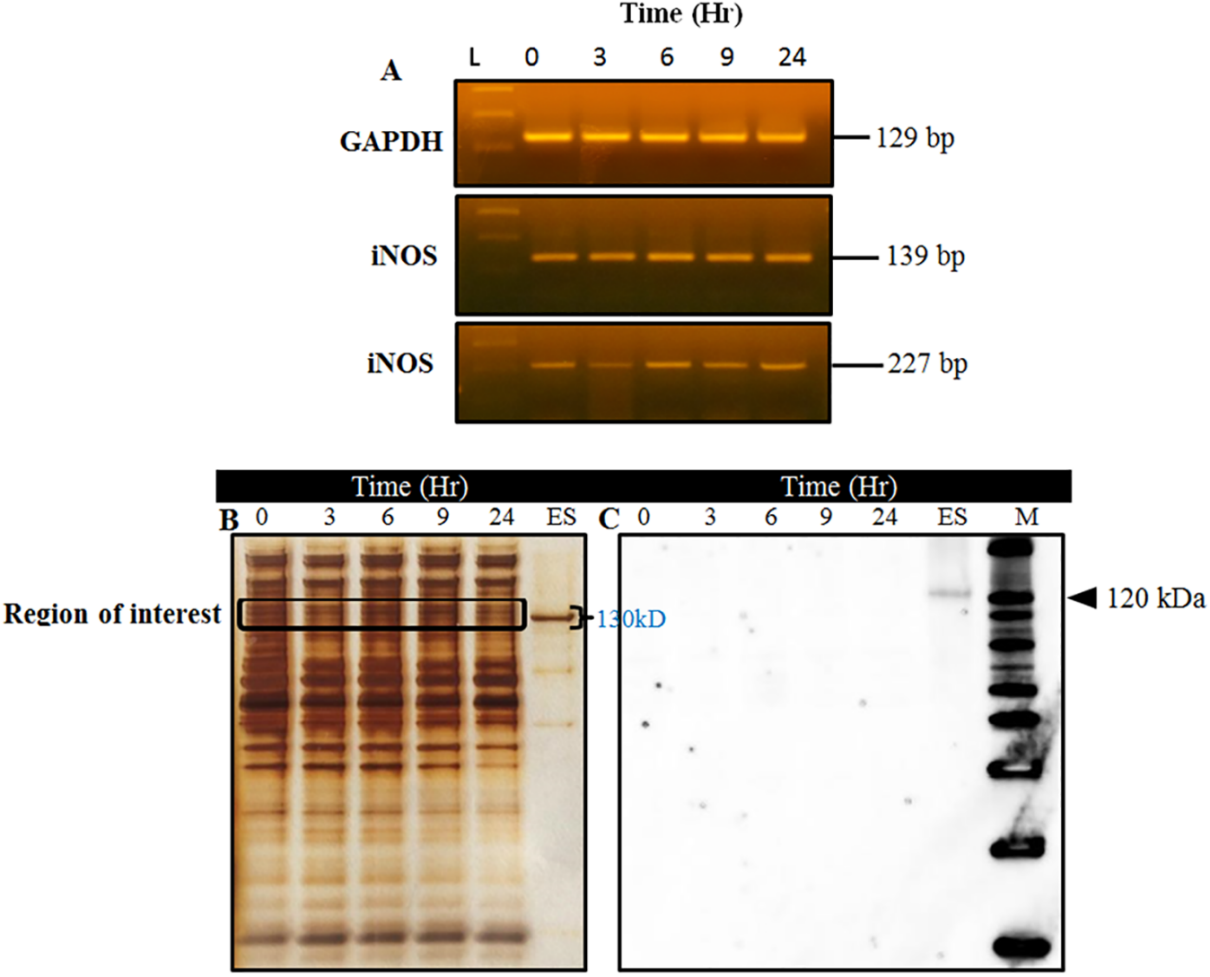
iNOS protein is recalcitrant to IL-1β-induction in canine articular chondrocytes. (A) iNOS mRNA expression in chondrocytes (P1) stimulated with 20 ng/mL of rhIL-1β for 0, 3, 6, 9 and 12 hr, (B) 20 μg protein from each treatment time was denatured and separated on a 12% SDS page and silver stained to visualize the profile of separated proteins, (C) iNOS protein levels were undetectable by Western blot, an indication of resistance to cytokine induction. L; 100bp DNA ladder, ES; iNOS Electrophoresis Standard ~130 kDa (loaded at 100 ng). M; protein standards marker, results shown are from one representative experiment of three (n = 3) independent experiments.

Due to nonspecific PCR products or dimer formation of iNOS primers designed earlier in the study, two sets of primers targeting two different regions of the 4.0 kbp iNOS mRNA were used to optimize the accuracy of the results. Treatment of chondrocytes with rhIL-1β resulted in upregulation iNOS, c-Jun, HIF-1α and HIF-2α mRNA expression. However, the preincubation of chondrocytes with PPS inhibited rhIL-1β-induced iNOS, c-Jun and HIF-1α mRNA upregulation (Figs 1A and 2B).

We observed a dose-dependent inhibitory effect of PPS on rhIL-1β-induced iNOS, c-Jun and HIF-1α mRNA upregulation (Fig 1B). Significant inhibition (*P* < 0.05) of iNOS mRNA was observed at PPS concentrations of 15 and 40 μg/mL compared to the positive control (PC), with the mRNA levels reaching levels similar to control chondrocytes (CTL, untreated). The expression of c-Jun and HIF-1α mRNA were significantly inhibited (*P* < 0.05) at almost all PPS concentrations compared to the PC (Fig 1B). However, HIF-2α mRNA remained relatively upregulated at 1, 5 and 15 μg/mL of PPS and was only significantly inhibited at 40 μg/mL compared to the PC (Fig 1B).

### iNOS protein is recalcitrant to cytokine induction; HIF-α proteins are undetectable under normoxia culture

While iNOS mRNA levels were readily detectable and upregulated by 20 ng/mL of rhIL-1β in cultured canine chondrocytes, by Western blot, the active iNOS protein levels were undetectable in all the lysed cells protein extracts at all stimulation time points (Fig 2A–2C).

**Fig 3:**
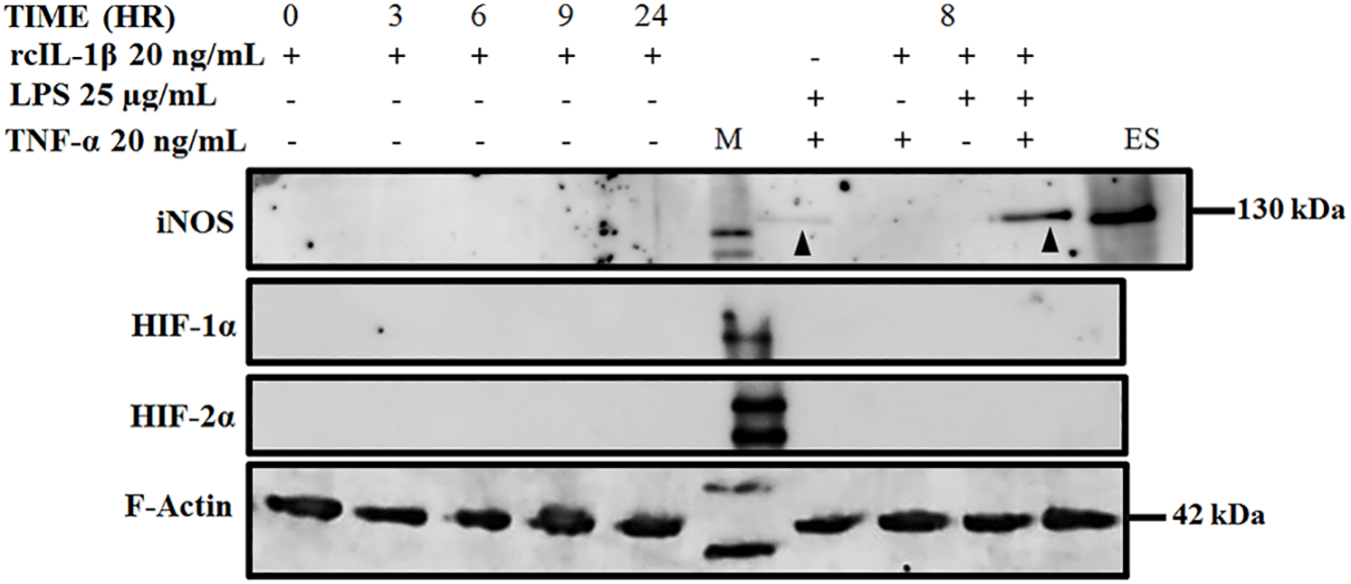
iNOS protein is recalcitrant to single or multiple cytokine induction. iNOS protein levels were undetectable in chondrocytes stimulated with single rcIL-1β, TNF-α or LPS. A combination of rcIL-1β + TNF-α + LPS induced a strong iNOS protein expression while a combination of TNF-α + LPS induced a very weak iNOS protein band as indicated by black arrowheads. HIF-1α and HIF-2α proteins were undetectable. ES; iNOS Standard protein ~130 kDa (loaded at 500 ng), M; Protein Standards marker. F-Actin (Internal control): 42 kDa, iNOS: 130 kDa, HIF-1α: 120 kDa and HIF-2α: 115 kDa. Blots shown are from one representative experiment of three (n = 3) independent experiments.

Surprisingly, similar to observations made with single rhIL-1β-stimulation, iNOS protein was still undetectable even in chondrocytes stimulated with single rcIL-1β, TNF-α or LPS including to a combination of rcIL-1β + TNF-α and rcIL-1β + LPS (Fig 3). Interestingly, stimulation of chondrocytes with a combination of TNF-α + LPS and rcIL-1β + TNF-α + LPS resulted in a weak and strong iNOS protein signal, respectively (Fig 3).

While the HIF-α isoforms were readily detectable at the mRNA level, both isoforms were undetectable at the protein level (Fig 3). Treatment of chondrocytes with single or multiple cytokines with or without LPS did not result in stabilization and detection of the HIF-α isoforms at protein level, an indication of rapid post-translational proteosomal degradation of the proteins in the presence of sufficient oxygen (Fig 3).

### PPS inhibits rcIL-1β + TNF-α + LPS-induced iNOS protein expression

**Fig 4:**
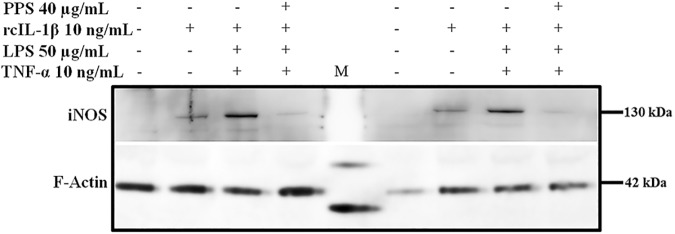
PPS inhibits rcIL-1β + TNF-α + LPS-induced iNOS protein expression. Single rcIL-1β (10 ng/mL) induced a weak iNOS protein signal compared to a combination of rcIL-1β (10 ng/mL) + TNF-α (10 ng/mL) + LPS (50 μg/mL) which significantly induced iNOS protein expression in canine chondrocytes. Pre-incubation of canine chondrocytes with 40 μg/mL of PPS significantly inhibited rcIL-1β + TNF-α + LPS-induced iNOS protein expression. M; Protein Standards marker. F-Actin (Internal control): 42 kDa, iNOS: 130 kDa. Blots shown are from two representative experiments of three (3) experiments.

Because iNOS protein expression was only significantly induced in chondrocytes treated with a combination of rcIL-1β + TNF-α + LPS, to confirm whether the observed inhibitory effects of PPS on iNOS mRNA were consistent with iNOS protein expression, 1.0 x 10^6^ chondrocytes were incubated as previously described. Thereafter, chondrocytes were treated with SF DMEM (CTL), 10 ng/mL of rcIL-1β, a combination of rcIL-1β (10 ng/mL) + TNF-α (10 ng/mL) + LPS (50 μg/mL) or preincubated with 40 μg/mL PPS for 4 hr then treated with a combination of rcIL-1β (10 ng/mL) + TNF-α (10 ng/mL) + LPS (50 μg/mL) for 8 hr. As consistently observed, a combination of rcIL-1β + TNF-α + LPS significantly induced iNOS protein expression but preincubation of chondrocytes with PPS significantly abrogated iNOS protein induction hence proving the inhibitory effects of PPS on both iNOS mRNA and protein expression (Fig 4).

### Immunocytochemistry: PPS colocalizes with NFkB and c-Jun

**Fig 5:**
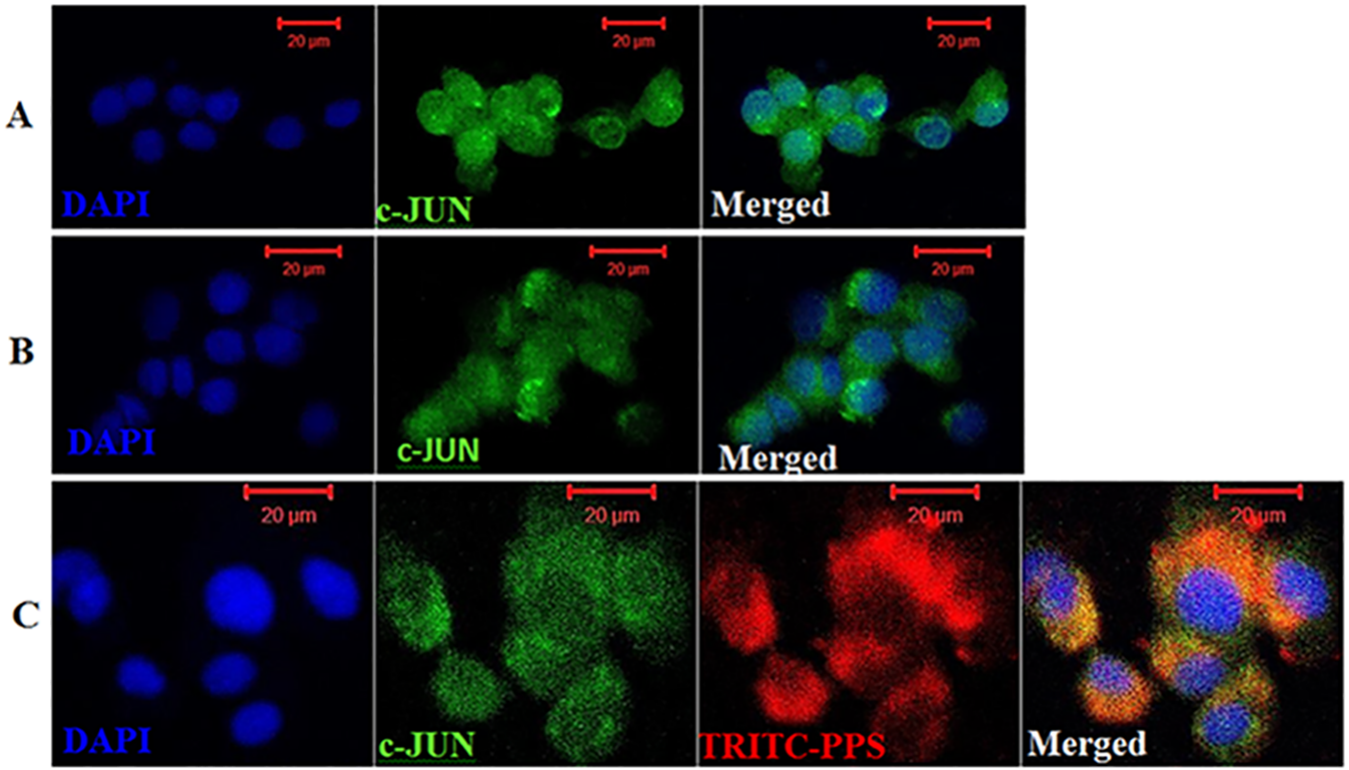
c-Jun colocalizes with pentosan polysulfate in canine articular chondrocytes. (A) Immunofluorescence staining of methanol-fixed chondrocytes showing diffuse nuclear accumulation of c-Jun (green) in control cells without rcIL-1β, (B) shows increased focal nuclear localization of c-Jun in chondrocytes stimulated with 10 ng/mL rcIL-1β (positive control, PC), and (C) shows chondrocytes preincubated with 40 μg/mL TRITC-PPS (red) for 4 hr then stimulated with 10 ng/mL of rcIL-1β. Chondrocytes preincubated with TRITC-PPS show reduced nuclear localization of c-Jun (green) indicated by increased perinuclear and cytoplasmic accumulation with TRITC-PPS (red) also accumulating in the perinuclear and cytoplasmic area. The yellow/orange areas in the merged image demonstrate that c-Jun colocalizes with PPS. **Scale Bars: 20** μ**M**

PPS [30,38–40] and other active glycosaminoglycans (GaGs) [41,42] have been proposed to exert their action through interaction with transcription factors and other intracellular proteins consequently blocking their downstream activity. ICC was performed to investigate colocalization of PPS with NFkB p65 and c-Jun, and to clarify whether PPS could inhibit NFkB p65 and c-Jun nuclear translocation and localization. Chondrocytes treated with or without 10 ng/mL of rcIL-1β predominantly showed nuclear localization of c-Jun (Fig 5A and 5B). However, when preincubated with 40 μg/mL of TRITC-PPS then treated with 10 ng/mL of rcIL-1β, chondrocytes demonstrated reduced c-Jun nuclear translocation (Fig 5C) as indicated by increased perinuclear and cytoplasmic localization. The merged image (yellow/orange areas) in Fig 5C indicates that c-Jun colocalizes with PPS.

**Fig 6:**
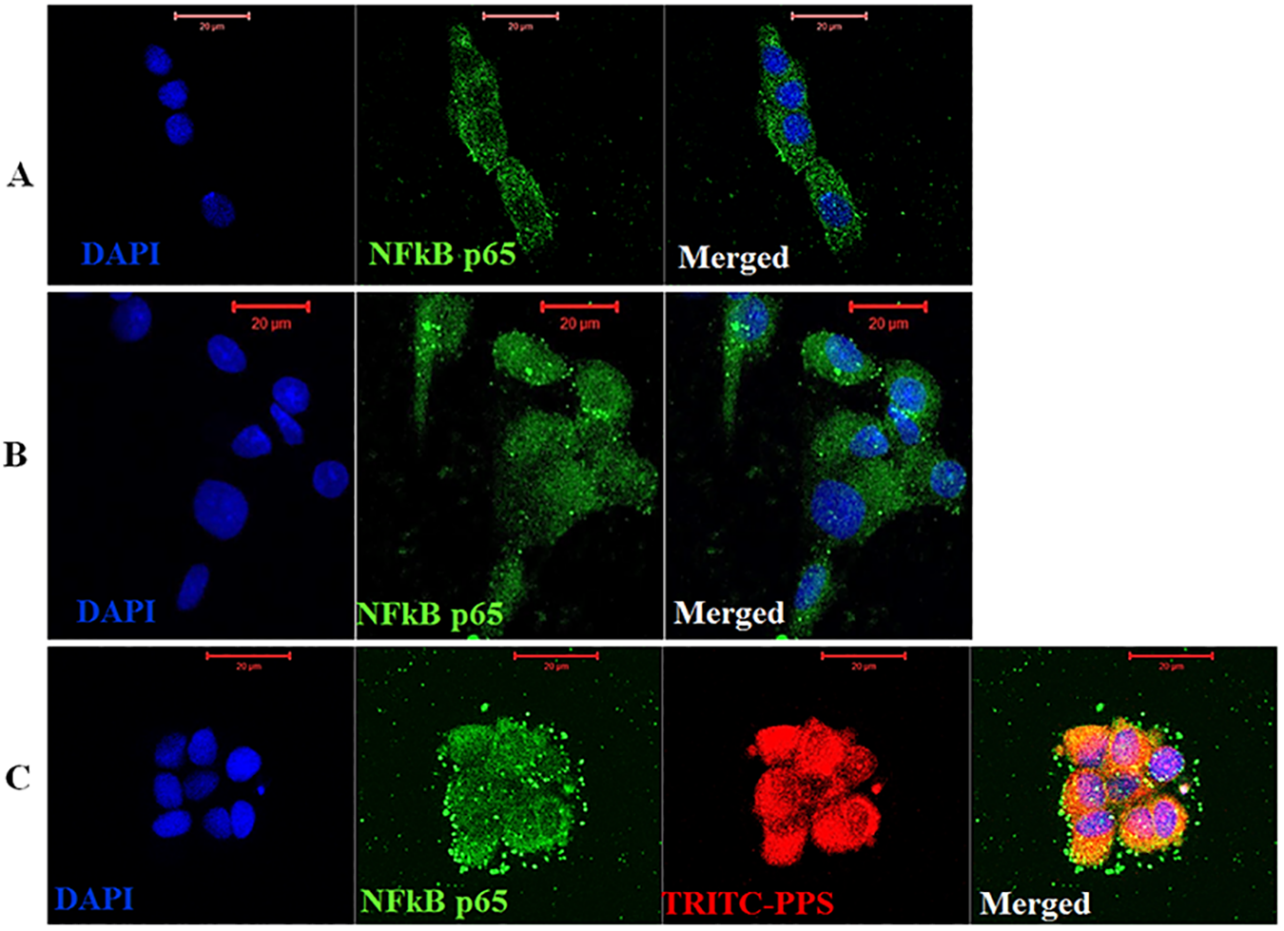
NFkB p65 colocalizes with pentosan polysulfate in canine articular chondrocytes. (A) Immunofluorescence staining of methanol-fixed chondrocytes without rcIL-1β (negative control) showing diffuse cytoplasmic localization of NFkB p65 (green), (B) shows increased nuclear localization and accumulation of NFkB p65 (green) in chondrocytes treated with 10 ng/mL of rcIL-1β (positive control, PC) and, (C) demonstrates that chondrocytes preincubated with 40 μg/mL of TRITC-PPS (red) for 4 hr then treated with 10 ng/mL of rcIL-1β have reduced NFkB p65 nuclear translocation activity as evidenced by increased cytoplasmic accumulation. The yellow/orange areas in the merged image show that NFkB p65 colocalizes with PPS. **Scale Bars: 20** μ**M**.

NFkB p65 predominantly accumulated in the cytoplasm of control chondrocytes without rcIL-1β (Fig 6A). As expected, treatment of chondrocytes with 10 ng/mL of rcIL-1β activated NFkB p65 as evidenced by increased nuclear translocation and localization (Fig 6B). However, preincubation of chondrocytes with 40 μg/mL of TRITC-PPS decreased rcIL-1β-induced NFkB p65 nuclear translocation in many chondrocytes as indicated by the accumulation and localization of the transcriptional factor in the perinuclear and cytoplasmic area (Fig 6C). The merged image of Fig 6C demonstrates that NFkB p65 colocalizes with PPS (yellow/orange areas) (Fig 6C).

## Discussion

The selective inhibition of pathologically enhanced NO synthesis by iNOS in OA joints is a novel therapeutic target for the treatment of OA [6,9,12]. The present study demonstrates for the first time that PPS is a novel inhibitor of IL-1β-induced iNOS, c-Jun and HIF-1α mRNA upregulation with limited inhibitory effect on IL-1β-induced HIF-2α upregulation. Similar to observations with normal human articular chondrocytes cultures [43,44], our finding demonstrates that normal CACs may be recalcitrant to single or multiple cytokine iNOS protein induction. While single IL-1β did not induce iNOS protein expression, treatment of CACs with a combination of rcIL-1β + TNF-α + LPS significantly induced iNOS protein expression but this effect was significantly abrogated by PPS hence proving its inhibitory effects on induced iNOS mRNA and protein expression. The detection of iNOS mRNA in monolayer cultured chondrocytes treated with or without IL-1β without active iNOS protein suggests that iNOS in CACs may be negatively regulated post-transcriptionally. Although more studies are required to fully elucidate the mechanism of action of PPS, ICC colocalization analysis results show that PPS colocalizes with NFkB and c-Jun, and inhibits their nuclear translocation and localization. This observation may partially support the suggestion that PPS may exert its inhibitory effects on OA by direct interaction with NFkB p65 and c-Jun, consequently repressing the downstream target genes like iNOS and MMP-13 which are implicated in the progression and perpetuation of OA.

While we observed IL-1-induced iNOS mRNA upregulation, the most provocative finding of the study was the undetectable iNOS protein in chondrocytes treated with single or multiple cytokines except to a combination of TNF-α + LPS (weak induction) and rcIL-1β + TNF-α + LPS (strong induction). Similar to our finding, normal human chondrocytes are reported to be recalcitrant to induction of iNOS even with multiple cytokines compared to all murine cell types in culture that exhibit a readily inducible iNOS [43,44]. This finding could suggest that normal CACs are recalcitrant to iNOS protein induction probably as a protective response from the deleterious effects of pathologically enhanced NO due to iNOS especially in the early phase of OA. Furthermore, this outcome may suggest that other than proinflammatory cytokines, other factors such as mechanical stress may play a pivotal role in iNOS protein induction in OA joints. Part of the signalling pathway cascade that leads to IL-1-induced NFkB activation and iNOS expression include the protein tyrosinase kinase (PTK) and p38. However, NFkB activation which is essential for iNOS induction and p38 lie on two distinct pathways that seem to be independently required for IL-1-induced iNOS expression [9,10]. While our laboratory previously demonstrated the inhibitory effects of PPS on the two pathways [30], by ICC, the present study confirms that indeed PPS impairs IL-1-induced NFkB p65 nuclear translocation but most interestingly we demonstrate that PPS colocalizes with NFkB p65. Therefore, the observed inhibition of IL-1-induced iNOS mRNA upregulation and rcIL-1β + TNF-α + LPS-induced iNOS protein expression by PPS is consistent with the inhibition of the NFkB pathway. Although more experiments such as immunoprecitiation assays will be required to prove the binding of PPS to transcription factors and other intracellular proteins, the observed colocalization with NFkB may support the proposal that it may exert its action through direct interaction with transcription factors consequently blocking their downstream promoter activity [30,38–40]. The detection of iNOS at mRNA level in monolayer cultured CACs in the absence IL-1β observed in this study was unusual and in contrast with previous studies in mouse [45], bovine [9,10], equine [15] including some isolated human chondrocytes [46,47]. In order to confirm whether iNOS mRNA is normally detectable in normal cultured CACs, we assessed its expression in chondrocytes derived from two other dogs cultured in medium without IL-1 and passaged up to P3. We verified the detection of iNOS mRNA in all passages (P0—P3) of CACs evaluated with low FBS concentration appearing to enhance its expression (S1 Fig). Whether the detection of iNOS at mRNA level in normal chondrocytes could be related to the dedifferentiation of chondrocytes in monolayer culture remains to be determined since epigenetic “unsilencing” of iNOS gene due to loss of DNA methylation at specific CpG sites in human OA chondrocytes has been associated with iNOS expression [43,48–50]. However, these findings taken together suggest that iNOS in CACs may be strongly regulated by translational blockade possibly via microRNA translational blockade which unlike in human cells has been reported to be strongly regulated at transcriptional level [10,44,51].

As part of cellular mediators of signal transduction, c-Jun together with c-FOS and ATF-2 (activating transcription factor) subunits form the complex dimeric transcription factors of activating protein-1 (AP-1) that bind to a common DNA site [52,53]. AP-1 just like NFkB seem to play a major role in mediating IL-1-induced early cellular responses [9,10,30]. While AP-1 is not required for IL-1-induced iNOS expression in articular chondrocytes, it is strongly activated by IL-1 [9,10] and its activation is required for the expression of MMPs, such as collagenase (MMP-13) and stromelysin (MMP-3), which promote cartilage degradation [10,54]. Particularly, JNK pathway has been shown to mediate the activation and transcription of c-Jun which is required for IL-1-induced MMP-13 upregulation [31] making c-Jun a potential therapeutic target for OA treatment. The results of this study demonstrate that IL-1β-induced c-Jun mRNA upregulation is inhibited by PPS. Our laboratory previously showed that PPS has no inhibitory effect on JNK activation but on ERK and p38 through inhibition of phosphorylation of these MAPKs [30]. Therefore, it is very reasonable to suggest that the observed inhibition of c-Jun mRNA by PPS could be an indication of PPS ability to directly interact with JNK thereby impairing the activation of c-Jun. Our ICC results also clearly demonstrate that PPS could impair IL-1-induced c-Jun nuclear translocation and localization in chondrocytes which may subsequently repress its MMP-13 promoter activity.

Consistent with results in human articular chondrocytes, our results confirm that both HIF-α isoforms in monolayer CACs are constitutively expressed at the mRNA level but are not readily detectable at protein level under normoxic culture condition as they are rapidly degraded in the presence of sufficient oxygen [33–37]. While both HIF-1α and HIF-2α mRNA were upregulated by IL-1β-stimulation, the preincubation of chondrocytes with PPS inhibited the IL-1β-induced upregulation of HIF-1α in a dose-dependent manner whereas minimal inhibitory effect on HIF-2α was observed except at the highest dose (40 μg/mL). Both HIF-1α and HIF-2α have been shown to be significantly overexpressed in cytoplasmic and nuclear of synovial lining and stromal cells derived from rheumatoid arthritis (RA) and OA human patients relative to cells from normal nonarthritic cases [55] and our regression analysis results also demonstrated a significant positive correlation of HIF-α isoforms expression in response to IL-1 (S2 Fig). HIF-1α levels in chondrocytes have been shown in part to be regulated by the NFkB and p38 mediated pathways such that inhibitors of NFkB and p38 significantly abolish IL-1 or TNF-α-induced HIF-1α upregulation [34,37,56]. Therefore the observed inhibitory effect of PPS on IL-1β-induced HIF-1α mRNA upregulation observed in this study is consistent with NFkB and p38 inhibition. HIF-1α mRNA has been shown to be higher in degenerated regions than in the intact regions of human OA articular cartilages a finding which was associated with cellular response to catabolic stress aimed at production of anti-apoptotic factors or act as a chondroprotective factor to maintain chondrocytes viability [56]. In confirming the anti-catabolic effects of IL-1 upregulated HIF-1α, another study using human articular chondrocytes cultured under hypoxic conditions, established these to be decreased cartilage degradation and MMP-13 expression [34]. On the other hand, HIF-2α has been proposed as the most potent transactivator that enhances the promoter activities MMP-13 and iNOS including other catabolic genes involved in OA process [19–21]. HIF-2α mRNA and protein expression was found to be enhanced in P0 cultures of mouse articular chondrocytes stimulated with proinflammatory cytokines including IL-1β, IL-17, IL-21 and TNF-α, and in human and mouse OA cartilage with its ectopic expression shown to trigger articular cartilage destruction in mice and rabbits [21]. While we observed a significant positive correlation between HIFs and iNOS mRNA expression, the present study does not prove any direct regulation of iNOS by HIFs, and in particular HIF-2α since the active protein was also undetectable under normoxic culture conditions to correlate with iNOS mRNA transcription and translation. Moreover, in the present study we detected iNOS protein without detectable HIF-2α protein levels. Contrary to the proposed catabolic effects, under hypoxic culture condition, human articular chondrocytes have been confirmed to increase tissue production via HIF-2α and inhibit cartilage destruction largely through HIF-1α [34]. These findings taken together demonstrate that HIF-2α mRNA may be upregulated in response to IL-1 but this response may also be corresponding to IL-1-upregulated HIF-1α since it has also been shown to be a potent regulator of autophagy in maturing mouse and human chondrocytes by acting as a brake to the autophagy accelerator function of HIF-1α [23]. Whether the overexpressed HIF-2α associated with induction of catabolic genes is due to its direct regulation of these genes leading to cartilage degradation or is related to its functional dysregulation in OA process remains to be verified.

In conclusion, to the best of our knowledge this is the first study to demonstrate that PPS is a novel inhibitor of IL-1-induced iNOS, c-Jun and HIF-1α mRNA upregulation and rcIL-1β + TNF-α + LPS-induced iNOS protein expression in CACs. In particular, the inhibitory effects of PPS on iNOS and c-Jun in articular chondrocytes could translate to its beneficial effects in the prevention and treatment of OA joints. Furthermore, our results show that normal CACs may be recalcitrant to single or multiple cytokine-induction of iNOS protein possibly as a protective mechanism to the deleterious effects of pathologically enhanced NO produced by iNOS.

## Supporting information

S1 Fig iNOS mRNA is detectable in monolayer passaged canine articular chondrocytes (CACs). Primary (P0) canine articular chondrocytes were cultured in 10% DMEM and 1% DMEM (P2^a^) and passaged up to third passage (P0 to P3). Cells were evaluated for iNOS mRNA expression by targeting the upstream 139bp and the downstream 227bp mRNA segments.
(TIF)

S2 Fig Regression line plot showing the correlation between HIF-α subunits and iNOS mRNA expression. (**A**) HIF-1α correlation with iNOS, (**B**) HIF-2α correlation with iNOS, and (**C**) HIF-1α correlation with HIF-2α. 95% confidence interval (95% CI); 0.53–1.05 (HIF-1α and iNOS), 0.41–0.81 (HIF-2α and iNOS) and 0.43–0.72 (HIF-1α and HIF-2α). Significant correlation defined as *P < 0.05, **P < 0.01. All the correlation analysis showed a strong significant correlation (*P* < 0.001) between the genes.
(TIF)

## References

[1] Krasnokutsky S, Attur M, Palmer G, Samuels J, Abramson SB. Current concepts in the pathogenesis of osteoarthritis. Osteoarthr Cartil. 2008;16 Suppl 3: S1–3. 10.1016/j.joca.2008.06.025 18723377

[2] Hellio Le Graverand-Gastineau M-P. OA clinical trials: current targets and trials for OA. Choosing molecular targets: what have we learned and where we are headed? Osteoarthr Cartil. 2009;17: 1393–1401. doi: 10.1016/j.joca.2009.04.009 19426849 10.1016/j.joca.2009.04.009

[3] MacPhail CM. Treatment of canine osteoarthritis. Waltham focus. 2000;10: 25–31.

[4] Murrell GA, Jang D, Williams RJ. Nitric oxide activates metalloprotease enzymes in articular cartilage. Biochem Biophys Res Commun. 1995;206: 15–21. doi: 10.1006/bbrc.1995.1003 7529496 10.1006/bbrc.1995.1003

[5] Järvinen TA, Moilanen T, Järvinen TL, Moilanen E. Nitric oxide mediates interleukin-1 induced inhibition of glycosaminoglycan synthesis in rat articular cartilage. Mediators Inflamm. 1995;4: 107–111. doi: 10.1155/S0962935195000184 18475625 10.1155/S0962935195000184 PMC2365616

[6] Stichtenoth DO, Frölich JC. Nitric oxide and inflammatory joint diseases. Br J Rheumatol. 1998;37: 246–257. 9566663 10.1093/rheumatology/37.3.246

[7] Zhong H, Ding Q, Chen W, Luo R. Vorinostat, a HDAC inhibitor, showed anti-osteoarthritic activities through inhibition of iNOS and MMP expression, p38 and ERK phosphorylation and blocking NF-κB nuclear translocation. Int Immunopharmacol. 2013;17: 329–335. doi: 10.1016/j.intimp.2013.06.027 23856614 10.1016/j.intimp.2013.06.027

[8] Boileau C, Martel-Pelletier J, Moldovan F, Jouzeau J-Y, Netter P, Manning PT, et al The in situ up-regulation of chondrocyte interleukin-1-converting enzyme and interleukin-18 levels in experimental osteoarthritis is mediated by nitric oxide. Arthritis Rheum. 2002;46: 2637–2647. doi: 10.1002/art.10518 12384922 10.1002/art.10518

[9] Mendes AF, Caramona MM, Carvalho AP, Lopes MC. Role of mitogen-activated protein kinases and tyrosine kinases on IL-1-Induced NF-kappaB activation and iNOS expression in bovine articular chondrocytes. Nitric Oxide. 2002;6: 35–44. doi: 10.1006/niox.2001.0378 11829533 10.1006/niox.2001.0378

[10] Mendes AF, Carvalho AP, Caramona MM, Lopes MC. Role of nitric oxide in the activation of NF-kappaB, AP-1 and NOS II expression in articular chondrocytes. Inflamm Res. 2002;51: 369–375. 12146729 10.1007/pl00000317

[11] Fernandes JC, Martel-Pelletier J, Pelletier J-P. The role of cytokines in osteoarthritis pathophysiology. Biorheology. 2002;39: 237–246. 12082286

[12] Balaganur V, Pathak NN, Lingaraju MC, More AS, Latief N, Kumari RR, et al Chondroprotective and anti-inflammatory effects of S-methylisothiourea, an inducible nitric oxide synthase inhibitor in cartilage and synovial explants model of osteoarthritis. J Pharm Pharmacol. 2014;66: 1021–1031. doi: 10.1111/jphp.12228 24697299 10.1111/jphp.12228

[13] Pelletier JP, Jovanovic D, Fernandes JC, Manning P, Connor JR, Currie MG, et al Reduced progression of experimental osteoarthritis in vivo by selective inhibition of inducible nitric oxide synthase. Arthritis Rheum. 1998;41: 1275–1286. doi: 10.1002/1529-0131(199807)41:7<1275::AID-ART19>3.0.CO;2-T 9663486 10.1002/1529-0131(199807)41:7<1275::AID-ART19>3.0.CO;2-T

[14] Pelletier JP, Lascau-Coman V, Jovanovic D, Fernandes JC, Manning P, Connor JR, et al Selective inhibition of inducible nitric oxide synthase in experimental osteoarthritis is associated with reduction in tissue levels of catabolic factors. J Rheumatol. 1999;26: 2002–2014. 10493683

[15] Tung JT, Venta PJ, Caron JP. Inducible nitric oxide expression in equine articular chondrocytes: effects of antiinflammatory compounds. Osteoarthr Cartil. 2002;10: 5–12. doi: 10.1053/joca.2001.0476 11795978 10.1053/joca.2001.0476

[16] Taskiran D, Stefanovic-Racic M, Georgescu H, Evans C. Nitric oxide mediates suppression of cartilage proteoglycan synthesis by interleukin-1. Biochem Biophys Res Commun. 1994;200: 142–148. 7513156 10.1006/bbrc.1994.1426

[17] McCartney-Francis N, Allen JB, Mizel DE, Albina JE, Xie QW, Nathan CF, et al Suppression of arthritis by an inhibitor of nitric oxide synthase. J Exp Med. 1993;178: 749–754. 7688035 10.1084/jem.178.2.749 PMC2191124

[18] Sakurai H, Kohsaka H, Liu MF, Higashiyama H, Hirata Y, Kanno K, et al Nitric oxide production and inducible nitric oxide synthase expression in inflammatory arthritides. J Clin Invest. 1995;96: 2357–2363. doi: 10.1172/JCI118292 7593623 10.1172/JCI118292 PMC185887

[19] Saito T, Fukai A, Mabuchi A, Ikeda T, Yano F, Ohba S, et al Transcriptional regulation of endochondral ossification by HIF-2alpha during skeletal growth and osteoarthritis development. Nat Med. 2010;16: 678–686. doi: 10.1038/nm.2146 20495570 10.1038/nm.2146

[20] Saito T, Kawaguchi H. HIF-2α as a possible therapeutic target of osteoarthritis. Osteoarthr Cartil. 2010;18: 1552–1556. doi: 10.1016/j.joca.2010.10.006 20950696 10.1016/j.joca.2010.10.006

[21] Yang S, Kim J, Ryu J-H, Oh H, Chun C-H, Kim BJ, et al Hypoxia-inducible factor-2alpha is a catabolic regulator of osteoarthritic cartilage destruction. Nat Med. 2010;16: 687–693. doi: 10.1038/nm.2153 20495569 10.1038/nm.2153

[22] Murphy CL. HIF-2alpha—a mediator of osteoarthritis? Cell Res. 2010;20: 977–979. doi: 10.1038/cr.2010.99 20625379 10.1038/cr.2010.99

[23] Bohensky J, Terkhorn SP, Freeman TA, Adams CS, Garcia JA, Shapiro IM, et al Regulation of autophagy in human and murine cartilage: hypoxia-inducible factor 2 suppresses chondrocyte autophagy. Arthritis Rheum. 2009;60: 1406–1415. doi: 10.1002/art.24444 19404942 10.1002/art.24444 PMC2747039

[24] Lafont JE, Talma S, Murphy CL. Hypoxia-inducible factor 2alpha is essential for hypoxic induction of the human articular chondrocyte phenotype. Arthritis Rheum. 2007;56: 3297–3306. doi: 10.1002/art.22878 17907154 10.1002/art.22878

[25] Lafont JE, Talma S, Hopfgarten C, Murphy CL. Hypoxia promotes the differentiated human articular chondrocyte phenotype through SOX9-dependent and -independent pathways. J Biol Chem. 2008;283: 4778–4786. doi: 10.1074/jbc.M707729200 18077449 10.1074/jbc.M707729200

[26] Vaughan-Scott T, Taylor JH. The pathophysiology and medical management of canine osteoarthritis. J S Afr Vet Assoc. 1997;68: 21–25. 9186936 10.4102/jsava.v68i1.861

[27] Richard F, Loeser J. Management of Osteoarthritis: Can We Slow Disease Progression ? Rev Artic. 2005;21: 104–106.

[28] Budsberg SC, Bergh MS, Reynolds LR, Streppa HK. Evaluation of pentosan polysulfate sodium in the postoperative recovery from cranial cruciate injury in dogs: a randomized, placebo-controlled clinical trial. Vet Surg. 2007;36: 234–244. doi: 10.1111/j.1532-950X.2007.00256.x 17461948 10.1111/j.1532-950X.2007.00256.x

[29] Innes JF, Barr AR, Sharif M. Efficacy of oral calcium pentosan polysulphate for the treatment of osteoarthritis of the canine stifle joint secondary to cranial cruciate ligament deficiency. Vet Rec. 2000;146: 433–437. 10811265 10.1136/vr.146.15.433

[30] Sunaga T, Oh N, Hosoya K, Takagi S, Okumura M. Inhibitory effects of pentosan polysulfate sodium on MAP-kinase pathway and NF-κB nuclear translocation in canine chondrocytes in vitro. J Vet Med Sci. 2012;74: 707–711. 22214865 10.1292/jvms.11-0511

[31] Mengshol JA, Vincenti MP, Coon CI, Barchowsky A, Brinckerhoff CE. Interleukin-1 induction of collagenase 3 (matrix metalloproteinase 13) gene expression in chondrocytes requires p38, c-Jun N-terminal kinase, and nuclear factor kappaB: differential regulation of collagenase 1 and collagenase 3. Arthritis Rheum. 2000;43: 801–811. doi: 10.1002/1529-0131(200004)43:4<801::AID-ANR10>3.0.CO;2-4 10765924 10.1002/1529-0131(200004)43:4<801::AID-ANR10>3.0.CO;2-4

[32] Freshney IR. Culture of animal cells; A manual of basic technique and specialized applications 6th ed. New Jersey: Wiley-Blackwell; 2010.

[33] Thoms BL, Murphy CL. Inhibition of hypoxia-inducible factor-targeting prolyl hydroxylase domain-containing protein 2 (PHD2) enhances matrix synthesis by human chondrocytes. J Biol Chem. 2010;285: 20472–20480. doi: 10.1074/jbc.M110.115238 20404338 10.1074/jbc.M110.115238 PMC2898297

[34] Thoms BL, Dudek KA, Lafont JE, Murphy CL. Hypoxia promotes the production and inhibits the destruction of human articular cartilage. Arthritis Rheum. 2013;65: 1302–1312. doi: 10.1002/art.37867 23334958 10.1002/art.37867

[35] Jaakkola P, Mole DR, Tian YM, Wilson MI, Gielbert J, Gaskell SJ, et al Targeting of HIF-alpha to the von Hippel-Lindau ubiquitylation complex by O2-regulated prolyl hydroxylation. Science. 2001;292: 468–472. doi: 10.1126/science.1059796 11292861 10.1126/science.1059796

[36] Lafont JE, Talma S, Murphy CL. Hypoxia-inducible factor 2alpha is essential for hypoxic induction of the human articular chondrocyte phenotype. Arthritis Rheum. 2007;56: 3297–3306. doi: 10.1002/art.22878 17907154 10.1002/art.22878

[37] Coimbra IB, Jimenez SA, Hawkins DF, Piera-Velazquez S, Stokes DG. Hypoxia inducible factor-1 alpha expression in human normal and osteoarthritic chondrocytes. Osteoarthr Cartil. 2004;12: 336–345. doi: 10.1016/j.joca.2003.12.005 15023385 10.1016/j.joca.2003.12.005

[38] Ghosh P, Wu J, Shimmon S, Zannettino AC, Gronthos S, Itescu S. Pentosan polysulfate promotes proliferation and chondrogenic differentiation of adult human bone marrow-derived mesenchymal precursor cells. Arthritis Res Ther. 2010;12: R28 doi: 10.1186/ar2935 20167057 10.1186/ar2935 PMC2875662

[39] Sadhukhan PC, Tchetgen M-B, Rackley RR, Vasavada SP, Liou L, Bandyopadhyay SK. Sodium Pentosan Polysulfate Reduces Urothelial Responses to Inflammatory Stimuli Via an Indirect Mechanism. J Urol. 2002;168: 289–292. 12050558

[40] Ghosh P. The pathobiology of osteoarthritis and the rationale for the use of pentosan polysulfate for its treatment. Semin Arthritis Rheum. 1999;28: 211–267. 10073500 10.1016/s0049-0172(99)80021-3

[41] Loeser RF, Im H-J, Richardson B, Lu Q, Chubinskaya S. Methylation of the OP-1 promoter: potential role in the age-related decline in OP-1 expression in cartilage. Osteoarthr Cartil. 2009;17: 513–517. doi: 10.1016/j.joca.2008.08.003 18829350 10.1016/j.joca.2008.08.003 PMC2692619

[42] Campo GM, Avenoso A, Campo S, D’Ascola A, Traina P, Samà D, et al Glycosaminoglycans modulate inflammation and apoptosis in LPS-treated chondrocytes. J Cell Biochem. 2009;106: 83–92. doi: 10.1002/jcb.21981 19009563 10.1002/jcb.21981

[43] de Andrés MC, Imagawa K, Hashimoto K, Gonzalez A, Roach HI, Goldring MB, et al Loss of methylation in CpG sites in the NF-κB enhancer elements of inducible nitric oxide synthase is responsible for gene induction in human articular chondrocytes. Arthritis Rheum. 2013;65: 732–742. doi: 10.1002/art.37806 23239081 10.1002/art.37806 PMC3937961

[44] Korhonen R, Lahti A, Kankaanranta H, Moilanen E. Nitric oxide production and signaling in inflammation. Curr Drug Targets Inflamm Allergy. 2005;4: 471–479. 16101524 10.2174/1568010054526359

[45] Campo GM, Avenoso A, Campo S, D’Ascola A, Traina P, Samà D, et al Glycosaminoglycans modulate inflammation and apoptosis in LPS-treated chondrocytes. J Cell Biochem. 2009;106: 83–92. doi: 10.1002/jcb.21981 19009563 10.1002/jcb.21981

[46] Palmer RM, Hickery MS, Charles IG, Moncada S, Bayliss MT. Induction of nitric oxide synthase in human chondrocytes. Biochem Biophys Res Commun. 1993;193: 398–405. doi: 10.1006/bbrc.1993.1637 7684906 10.1006/bbrc.1993.1637

[47] Otero M, Lago R, Lago F, Reino JJG, Gualillo O. Signalling pathway involved in nitric oxide synthase type II activation in chondrocytes: synergistic effect of leptin with interleukin-1. Arthritis Res Ther. 2005;7: R581–591. doi: 10.1186/ar1708 15899045 10.1186/ar1708 PMC1174950

[48] Imagawa K, de Andrés MC, Hashimoto K, Pitt D, Itoi E, Goldring MB, et al The epigenetic effect of glucosamine and a nuclear factor-kappa B (NF-kB) inhibitor on primary human chondrocytes—implications for osteoarthritis. Biochem Biophys Res Commun. 2011;405: 362–367. doi: 10.1016/j.bbrc.2011.01.007 21219853 10.1016/j.bbrc.2011.01.007 PMC3937866

[49] Cheung KSC, Hashimoto K, Yamada N, Roach HI. Expression of ADAMTS-4 by chondrocytes in the surface zone of human osteoarthritic cartilage is regulated by epigenetic DNA de-methylation. Rheumatol Int. 2009;29: 525–534. doi: 10.1007/s00296-008-0744-z 18941754 10.1007/s00296-008-0744-z

[50] Roach HI, Yamada N, Cheung KSC, Tilley S, Clarke NMP, Oreffo ROC, et al Association between the abnormal expression of matrix-degrading enzymes by human osteoarthritic chondrocytes and demethylation of specific CpG sites in the promoter regions. Arthritis Rheum. 2005;52: 3110–3124. doi: 10.1002/art.21300 16200590 10.1002/art.21300

[51] Förstermann U, Kleinert H. Nitric oxide synthase: expression and expressional control of the three isoforms. Naunyn Schmiedebergs Arch Pharmacol. 1995;352: 351–364. 8532063 10.1007/BF00172772

[52] Karin M, Liu Z g, Zandi E. AP-1 function and regulation. Curr Opin Cell Biol. 1997;9: 240–246. 9069263 10.1016/s0955-0674(97)80068-3

[53] Whitmarsh AJ, Davis RJ. Transcription factor AP-1 regulation by mitogen-activated protein kinase signal transduction pathways. J Mol Med Berl Ger. 1996;74: 589–607. 10.1007/s001090050063 8912180

[54] Zafarullah M, Martel-Pelletier J, Cloutier JM, Gedamu L, Pelletier JP. Expression of c-fos, c-jun, jun-B, metallothionein and metalloproteinase genes in human chondrocyte. FEBS Lett. 1992;306: 169–172. 1633872 10.1016/0014-5793(92)80992-p

[55] Giatromanolaki A, Sivridis E, Maltezos E, Athanassou N, Papazoglou D, Gatter KC, et al Upregulated hypoxia inducible factor-1alpha and -2alpha pathway in rheumatoid arthritis and osteoarthritis. Arthritis Res Ther. 2003;5: R193–201. doi: 10.1186/ar756 12823854 10.1186/ar756 PMC165055

[56] Yudoh K, Nakamura H, Masuko-Hongo K, Kato T, Nishioka K. Catabolic stress induces expression of hypoxia-inducible factor (HIF)-1 alpha in articular chondrocytes: involvement of HIF-1 alpha in the pathogenesis of osteoarthritis. Arthritis Res Ther. 2005;7: R904–914. doi: 10.1186/ar1765 15987493 10.1186/ar1765 PMC1175045

