# Supplementary figures and images for: Pentosan polysulfate inhibits IL-1β-induced iNOS, c-Jun and HIF-1α upregulation in canine articular chondrocytes
Source: PLoS One. 2017 May 4;12(5):e0177144. doi: 10.1371/journal.pone.0177144 (PMC5417682; doi:10.1371/journal.pone.0177144)

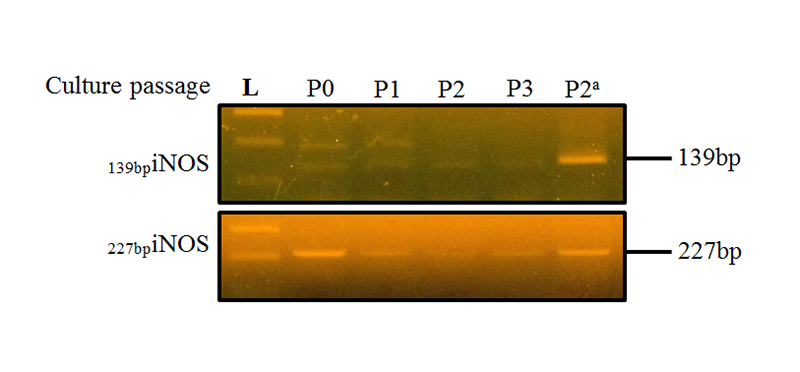

Supplement: S1 Fig — Primary (P0) canine articular chondrocytes were cultured in 10% DMEM and 1% DMEM (P2a) and passaged up to third passage (P0 to P3). Cells were evaluated for iNOS mRNA expression by targeting the upstream 139bp and the downstream 227bp mRNA segments. (TIF) [file pone.0177144.s001.tif]

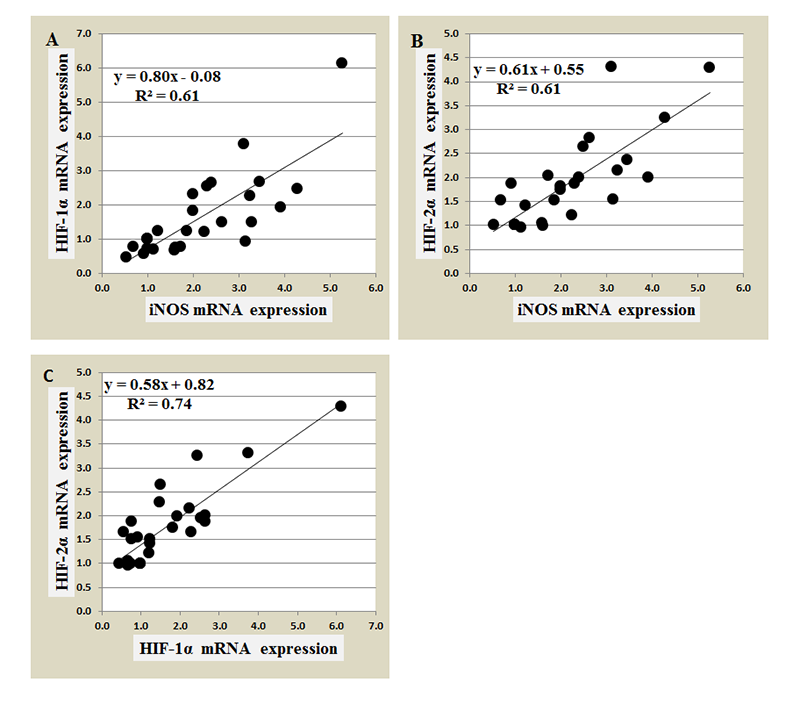

Supplement: S2 Fig — (A) HIF-1α correlation with iNOS, (B) HIF-2α correlation with iNOS, and (C) HIF-1α correlation with HIF-2α. 95% confidence interval (95% CI); 0.53–1.05 (HIF-1α and iNOS), 0.41–0.81 (HIF-2α and iNOS) and 0.43–0.72 (HIF-1α and HIF-2α). Significant correlation defined as *P < 0.05, **P < 0.01. All the correlation analysis showed a strong significant correlation (P < 0.001) between the genes. (TIF) [file pone.0177144.s002.tif]
